# Gonadotropin-Releasing Hormone (GnRH) Agonists Do Not Protect Ovarian Function in Patients Undergoing Stem Cell Transplants

**DOI:** 10.7759/cureus.58387

**Published:** 2024-04-16

**Authors:** Ariel Benor, Alan Decherney

**Affiliations:** 1 Reproductive Endocrinology and Infertility, National Institutes of Health, Bethesda, USA

**Keywords:** premature ovarian insufficiency, ovarian reserve, menstrual dysfunction, fertility sparing treatment, fertility preserving, stem-cell transplant, gonadoprotection, gonadotoxicity

## Abstract

Introduction

Medical indications for fertility preservation include any malignancy, chronic illness, or disease that would require gonadotoxic chemotherapy or radiation (conditioning regimens), which would impede a woman's ability to conceive in the future. Thus, any patient who plans to undergo a gonadotoxic regimen is advised to cryopreserve oocytes or embryos, which can be used in the future at the patient's convenience. Attempts have been made to suppress ovarian function with gonadotropin-releasing hormone agonists (GnRH-a) to induce ovarian quiescence and, thereby, theoretically limit the gonadotoxic impact on the follicular pool. We explored the use of leuprolide (a type of GnRH-a) in preventing primary ovarian insufficiency (POI) in a cohort study of patients who underwent hematopoietic stem cell transplants (HSCT) at the National Institutes of Health (NIH); since the conditioning regimens for HSCT include cyclophosphamide and other gonadotoxic therapies, we hypothesized that GnRH-a would be ineffective in preventing POI.

Methods

We assessed patients who underwent fertility preservation prior to their stem cell transplant, as their follicular-stimulating hormone (FSH) levels were evaluated prior to and post-chemotherapy. Twenty-nine patients who underwent hormonal evaluation prior to and post-chemotherapy were included. The control group did not receive GnRH-a prior to chemotherapy, while the treatment group did receive GnRH-a pre-chemotherapy.

Results

Our data revealed that 80% of the control group had menopausal levels post-chemotherapy, while 91% of the treatment group still had menopausal levels post-chemotherapy (p=0.33).

Conclusions

Thus, our hypothesis that GnRH-a is ineffective in reducing the risk of POI in a cohort of patients who receive conditioning regimens for HSCT was confirmed.

## Introduction

Fertility preservation for medical indications is challenging in many regards. Women with medical conditions that warrant fertility preservation are often encumbered by limited time, resources, and finances, which can be significant barriers to freezing their oocytes for future use. In the realm of medically indicated fertility preservation, there is no consensus on whether the use of GnRH (gonadotropin-releasing hormone) agonists protects ovaries from the gonadotoxic effects of chemotherapy. There is conflicting data on the gonado-protective effect of “ovarian suppression,” a technique whereby iatrogenic suppression of the hypothalamic-pituitary-ovarian (HPO) axis purportedly protects the functional ovarian reserve from destruction by toxic agents. Some studies report a higher rate of return of menses after chemotherapy in patients who received a GnRH agonist (GnRH-a) compared to the controls [1-7]; however, there are data contradicting that thesis, asserting that the rates of ovarian insufficiency are similar [8-13]. Although iatrogenic POI (primary ovarian insufficiency) is a known sequela of gonadotoxic chemotherapy, the use of a GnRH-a to mitigate the destruction of the oocyte reserve by temporarily suppressing ovarian function has not been thoroughly corroborated. The American Society for Reproductive Medicine’s committee opinion clearly states: “GnRH agonists may be offered to breast cancer patients to reduce the risk of premature ovarian insufficiency but should not be used in place of other fertility preservation alternatives” [14]. This statement implies that solely using a GnRH-a as a means of preserving fertility is not recommended; moreover, it specifies the use in breast cancer patients only. However, its concomitant use as an adjuvant treatment, with the intended goal of inducing amenorrhea, is commonly utilized to thwart preventable sequela of chemotherapy, i.e. abnormal uterine bleeding secondary to thrombocytopenia [14]. Hence, GnRH-a is often administered to suppress the HPO axis prior to gonadotoxic agents, in order to induce endometrial atrophy and avoid iatrogenic uterine bleeding during an inauspicious period.

The studies referenced above have mostly assessed women undergoing chemotherapy for premenopausal breast cancer; there is limited data describing the effectiveness of gonado-protection, e.g. prevention of POI, in patients undergoing hematopoietic stem cell transplants (HSCT). Patients undergoing HSCT receive different chemotherapeutic agents than do patients with breast cancer; thus, the data from studies analyzing these outcomes in breast cancer patients cannot be extrapolated to HSCT cohorts. Moreover, most of the studies assess menstrual status as a marker of ovarian function, which may be unreliable due to recall bias and patient subjectivity.

Our objective was to assess whether the administration of a GnRH-a reduces the risk of POI in patients undergoing conditioning regimens for an HSCT. Our null hypothesis was that patients who received depot leuprolide (GnRH-a) injections would not have higher rates of menopausal follicular-stimulating hormone (FSH) levels than the controls.

Since all stem cell transplants necessitate some chemotherapy to ablate the immunocompetent cells of the host or to induce remission from malignancy, there have been attempts at minimizing the collateral damage to the oocyte reserve by administering reduced-intensity conditioning (RIC) regimens, which are less toxic to organ systems than myeloablative conditioning (MAC) regimens. Moreover, since pelvic radiation further diminishes the ovarian reserve, many conditioning regimens have recently been formulated to avoid radiation in premenopausal women who want to attempt to preserve their fertility. Unlike the chemotherapy used for breast cancer, the conditioning regimens for stem cell transplants utilize high doses of alkylating or other gonadotoxic agents; thus, the rates of ovarian insufficiency post-HSCT are significantly higher compared to those receiving chemotherapy for breast cancer [15]. Moreover, the diagnoses spurring an indication for HSCT vary from benign to malignant. Benign indications for HSCT include sickle cell disease and genetic mutations leading to immunodeficiency diseases, i.e. GATA2, STAT3, DOCK8, PI3K gene mutations. On the other hand, malignancies that are treated with HSCT include lymphomas (Hodgkin’s, B-cell), leukemia (myelogenous, lymphocytic, hairy cell), and myelodysplastic syndrome (MDS). The rates of POI vary based on the type of conditioning regimen (MAC or RIC) used as well as whether radiation is administered [2]. Transient, spurious menses in the setting of POI are not suggestive of normal ovarian function, although some studies evaluate menstrual function as a surrogate marker of resumption of premenopausal ovarian function [4-8].

Since most conditioning regimens for HSCT include some form of gonadotoxic agent, whether it be chemotherapy or radiation, our goal was to assess ovarian function post-HSCT in the treatment group, which received GnRH-a to induce ovarian quiescence, compared to the control group, which did not.

## Materials and methods

We aimed to describe the residual ovarian function in a cohort of patients who received gonadotoxic chemotherapy via the assessment of a pituitary hormonal assay, i.e. FSH levels, as a reflection of the functional ovarian reserve. This is a cohort study of women with chronic medical conditions who underwent chemotherapy in conjunction with a stem cell transplant at the National Institutes of Health (NIH). All these patients were on Institutional Review Board (IRB)-approved protocols, and only de-identified data was analyzed; therefore, the collection of this data was IRB-exempt. Patients from 2014 to 2021 with any medical condition that qualified them for an HSCT who were referred to the reproductive gynecology division at the NIH for fertility preservation beforehand were included in the analysis; patients were only included if they were premenopausal, i.e. normal menstrual function and basal FSH levels <30 IU/L in the early follicular phase. The electronic medical record database from our research-based institution was queried to obtain medical information from the patients who received an HSCT. Since this was a retrospective study, we only utilized information that was already available. Sixty-nine patients were initially included based on the inclusion criteria: age under 40 at the time of HSCT and having undergone fertility preservation. Twenty patients were excluded due to a lack of follow-up hormonal results post-chemotherapy. Forty-nine patients were included in the analysis.

All patients received some form of gonadotoxic agent as a conditioning regimen prior to the stem cell transplant, mainly cyclophosphamide, cytarabine, and busulfan. The cyclophosphamide equivalent dose (CED) was calculated for each patient. The gonadal function was assessed indirectly via FSH levels at various time points within two years after chemotherapy, as there is no standard time-point after chemotherapy for assessing the return of function; although most patients only had one FSH level measured post-chemotherapy, all FSH levels available were assessed to assess for return of normal function at later time points. An FSH level of 30 IU/L was used as the menopausal level in conjunction with four months of amenorrhea, concurrent with the American College of Obstetricians and Gynecologists' diagnosis of POI [16]. Additional FSH levels at further time points were assessed to evaluate for return of ovarian function. We utilized the STROBE (STrengthening the Reporting of OBservational studies in Epidemiology) checklist guideline for our cohort study, which is a standardized guideline for retrospective studies (see Appendices) [17].

The data was presented in ordinal scale (numerical values of FSH), yet we fashioned them into categorical data (menopausal levels being >30 IU/L vs. non-menopausal levels <30 IU/L) for our analysis. We compared the control group that did not receive GnRH-a prior to chemotherapy to the treatment group that did receive GnRH-a prior to chemotherapy. For our statistical analysis, we utilized Fisher's exact test since the study design is unpaired (the results of two groups of different sizes are compared). Because we compared the incidence of menopausal levels in two small groups, the Fisher exact test was most apt for our analysis. We performed our calculations using this website: https://www.socscistatistics.com/tests/fisher/default2.aspx and considered alpha <0.05 as significant.

## Results

The baseline demographics and hormonal assay results are presented in Table 1. The ages of the patients ranged from 13 to 37; 26 out of the 29 patients assessed were under age 30. The diagnoses of the patients included primary immunodeficiency diseases (DOCK8: n=4, STAT3: n=1, GATA2 mutations: n=11), lymphoma (n=3), leukemia (n=5), and sickle cell disease (n=5).

**Table 1:** Baseline demographics, hormonal/ovarian reserve parameters for treatment and control groups CED: cyclophosphamide equivalent dose; PID: primary immunodeficiency disease; HSCT: hematopoietic stem cell transplant; FSH: follicular-stimulating hormone; E2: estradiol; AFC: antral follicle count; AMH: anti-Müllerian hormone *AFC was not recorded for one patient in the control and for four patients in the treatment group.

	Control (no leuprolide)	Treatment (leuprolide)
Mean age, y; (std. dev.)	26.6 (7.6)	21.7 (5.9)
Age range	20-37	13-33
Diagnosis (n)	5	24
Sickle cell disease (n)	4	1
Leukemia	1	3
Lymphoma	0	3
PID	0	17
CED, mean (mg/m^2^); (SD)	3299.0 (2937.9)	3948.2 (2447.5)
# of pts. who received total body irradiation (n, %)	3 (60%)	4 (16.7%)
Pre-treatment FSH, mean (IU/L); (SD)	4.5 (1.5)	6.2 (3.0)
Pre-treatment E2, mean (pg/mL); (SD)	42.7 (29.6)	41.2 (22.4)
Post-HSCT FSH, mean	65.1 (14.3)	84.4 (18.4)
AFC, mean*; (SD)	14.8 (7.4)	18.4 (11.1)
Pre-treatment AMH, mean; (SD)	2.4 (1.6)	3.2 (3.0)
Gravidity prior to chemotherapy (n,%)		
Nulligravida	2 (40%)	23 (96%)
Primigravida	1 (20%)	0
Multigravida	2 (40%)	1 (4%)

**Figure 1:**
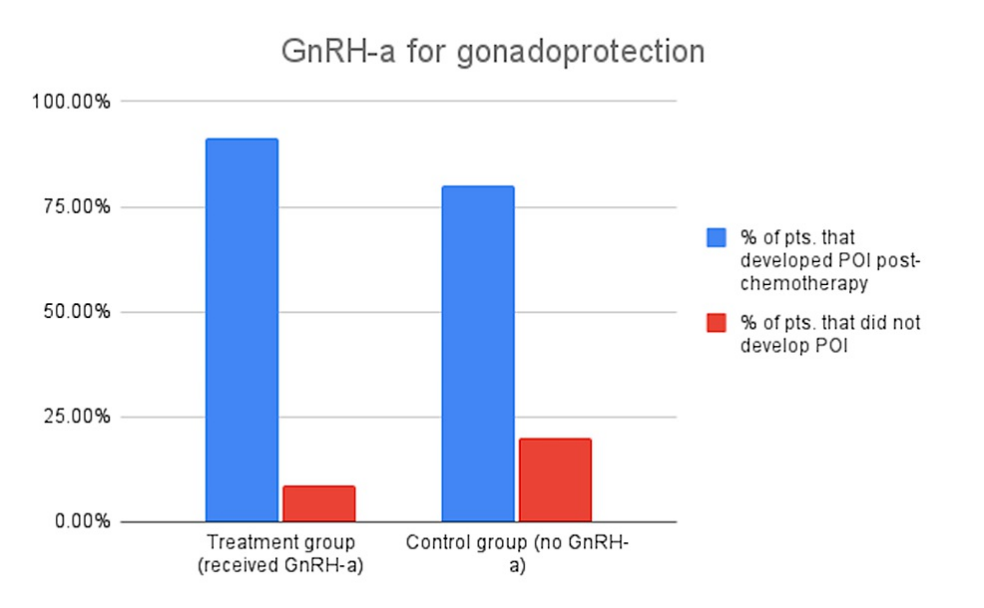
Graph of the percentage of patients who developed POI in treatment and control groups GnRH-a: gonadotropin-releasing hormone agonists; POI: primary ovarian insufficiency

Twenty-six out of 29 patients were nulliparous (two with sickle cell disease, one with leukemia). FSH levels were assessed prior to receiving chemotherapy and at least three months post-chemotherapy. The mean CED in the control group (no leuprolide) was 3299 mg/m^2^, while the mean CED in the treatment group (received leuprolide) was 3948 mg/m^2^. Twenty-four patients received depot leuprolide injections prior to chemotherapy, and five patients did not receive a depot leuprolide injection. Of the 24 patients who received the leuprolide, 23/24 (91.3%) had at least one FSH level in the menopausal range when assessed after chemotherapy. That patient was aged 13 with a DOCK8 gene mutation (PID); she received chemotherapy (CED of 1828 mg/m^2^) only eight months post-menarche, and she had an undetectable estradiol (E2) level and FSH level of 2.4 IU/L at a time-point 36 months post-HSCT, which connotes hypothalamic hypogonadism. Of the five patients who did not receive a depot leuprolide injection, four out of five (80%) had at least one menopausal FSH level. The outlier had a near-cutoff FSH value of 27.7 IU/L. The two groups’ post-transplant FSH levels were compared categorically: at least one menopausal FSH level (>30 IU/L) vs. no menopausal levels (<30 IU/L). A Fisher's exact test was run, and the p-value resulted as 0.33 (Figure 1). The result, thus, is not statistically significant at p<0.05.

Subsequently, additional FSH and E2 values were assessed at further time points to evaluate whether the function of time has a role in the recovery of ovarian status. There were 28 patients that had both hormonal assays (FSH, E2) evaluated at least one additional time-point post-HSCT. Two out of the five patients (40%) in the control group had a return of ovarian function with premenopausal FSH and E2 levels (<30 IU/L). In contrast, only four out of 23 patients (17.4%) in the treatment group had a return of premenopausal FSH and E2 levels. A Fisher's exact test was run, and the p-value resulted at 0.29; thus, it was not found to be statistically significant.

## Discussion

Patients with certain chronic illnesses, either due to malignancy, congenital immunodeficiency, or hemoglobinopathy, are often left with HSCT as the only curative treatment. Unfortunately, the conditioning regimen that is necessary to prime the host for transplant usually includes gonadotoxic therapies, which often leads to ovarian insufficiency [2,8]. Attempts have been made to protect the ovaries from toxic damage by suppressing their function temporarily with GnRH-a, which puts them into a quiescent state. Our study demonstrates no difference in gonadoprotection between the group that received GnRH-a prior to chemotherapy compared to the group that did not receive GnRH-a.

One group of chemotherapy, alkylating agents, are the most potent gonadotoxic agents. Cyclophosphamide, one type of alkylating agent, is the most gonadotoxic chemotherapeutic agent (via its metabolite phosphoramide mustard), yet there is a multitude of others that have a similar, albeit less pronounced, effect. Hence, the equivalency scale (CED) relates the toxicity of other types of chemotherapy to that of cyclophosphamide [18]. The alkylating agents do not alkylate DNA but rather cause covalent DNA adducts. They are not cell-cycle-specific, so a cell does not need to be actively replicating to be destroyed. These agents destroy pregranulosa cells of primordial follicles, which produce anti-Müllerian hormone (AMH) and support follicular growth and development [1-3,18].

There is controversial evidence regarding the gonado-protective effects of adjuvant GnRH-a therapy on ovarian function in patients undergoing gonadotoxic treatments. The prior studies investigating this effect have analyzed patients with varying diagnoses (mostly breast cancer and lymphoma), using a multitude of types of chemotherapy; since not all types affect the ovarian reserve to the same degree we calculated the CED in our patients [3-12].

For instance, Demesteere et al. examined the effects of GnRH-a on ovarian reserve markers (FSH, AMH) in 129 patients with lymphoma and concluded that it is not effective in preventing ovarian insufficiency [11]. Those patients received doxorubicin, bleomycin, vinblastine, and dacarbazine, of which only the latter is an alkylating agent. On the contrary, a retrospective study of 44 women with autoimmune disorders (connective tissue disease/mixed connective tissue disorder, systemic lupus erythematosus, systemic sclerosis, systemic vasculitis) demonstrated a protective effect of a GnRH-a on ovarian reserve parameters after receiving cyclophosphamide. However, the control group only had 11 patients, and the mean age of patients who developed ovarian insufficiency was 32 compared to 26 in the group that resumed ovarian function [3]. This study by Blumenfeld et al. clearly demonstrates that age, and therefore ovarian reserve, at the time of chemotherapy is an important prognostic indicator of the resumption of normal menstruation; chronic illness, inflammation, and the length of time a patient has had autoimmune flares may also be contributory [3].

Our results are consistent with an RCT by Elgindy et al. of 100 premenopausal breast cancer patients, which showed no differences in menstruation resumption rates or hormonal and ultrasound markers between the treatment (GnRH-a) and control groups [19]. Those women received 5-fluorouracil, adriamycin, and cyclophosphamide; there were no differences in age groups. Menstruation resumption rates did not differ at one year after chemotherapy; therefore, they concluded that GnRH-a did not have a protective effect on chemotherapy-induced gonadotoxicity. Our findings corroborate their data.

On the other hand, Sverrisdottir et al. performed a randomized controlled trial (RCT) of 285 premenopausal breast cancer patients and demonstrated that the group that received a GnRH-a had higher rates of resumption of menses compared to the control [6]. However, this study only assessed menstrual patterns as a surrogate marker, and the mean age of the patients was 45. The gonadotoxic effect on the ovarian reserve depends on the follicular pool present at the time of chemotherapy, as the response is proportional to the quantity of primordial follicles and granulosa cells present. The functional ovarian reserve depletes rapidly after age 40, so this cohort of patients is much older than ours. Therefore, Walshe et al. demonstrate that the risk of POI secondary to chemotherapy is proportional to a woman’s age, with the risk increasing at higher ages due to the depleting oocyte reserve in women as they age [20].

In a similar vein to age, the regimen and dosage of chemotherapy, the utilization of pelvic radiation, and the baseline ovarian reserve prior to gonadotoxic treatment are the key variables that factor into the risk of developing POI. Since ovarian reserve markers, i.e. AMH and FSH, reflect a woman’s follicular pool, they are the most common surrogate markers of functional ovarian reserve, as there is no feasible method of quantifying the number of remaining oocytes in a living person.

Many of the studies evaluating ovarian function post-chemotherapy assessed for menstrual status. For instance, an RCT by Munster et al. enrolled patients with stages I-III breast cancer under age 45 and assessed menstruation patterns after chemotherapy. They performed a proportional hazards model to determine whether a GnRH-a had any impact on the time to the resumption of menses after chemotherapy. The hazard ratio did not demonstrate any effect on the time to the resumption of menses [21]. Although there are no standard time points after chemotherapy for assessing functional ovarian reserve or return of function, FSH levels in that study were assessed in three intervals: first six months, 6-18 months, and >18 months post-chemotherapy. They determined that amenorrhea rates did not differ in the group that received GnRH-a compared to that of the control group. Our data corroborate those findings: time does not significantly contribute to the resumption of ovarian function after the toxic insult.

In another study by Foregeard et al., which assessed ovarian function after HSCT, 74% of the patients developed POI [22]. There was a significantly higher risk in those who received MAC regimens compared to those who received RIC regimens (47% of the patients who received RIC still developed POI). Moreover, the condition/diagnosis that necessitated the HSCT also correlated to the risk of POI in this study. Those patients with leukemia had an 81% rate of POI while the group with bone marrow failure developed POI at a rate of only 14% [22]. This alludes to three notions: (1) the length of time that a patient suffers from a chronic disease, (2) the etiology of the condition, and (3) the dosage of chemotherapy required may each contribute to the depletion of the ovarian reserve.

Our data corroborate previously published data that refutes the efficacy of GnRH-a in reducing the risk of developing POI after gonadotoxic chemotherapy. We clearly demonstrate here that in our cohort of patients who received conditioning regimens with alkylating agents in preparation for an HSCT, there was no difference in menopausal hormone levels between the treatment and control groups. This data may be generalizable only to patients who undergo conditioning regimens for HSCT and not to other chemotherapeutic regimens.

Our study has its limitations (small cohort, retrospective nature), but it adds to the scarce data about ovarian reserve parameters in patients who receive conditioning regimens for HSCT. Although the patients in our study received varying chemotherapeutic and radiation regimens, the strength of our study is that we calculated the CED for each patient, and there were no differences between the two groups. Moreover, we utilized the available FSH levels within a two-year time period post-chemotherapy as opposed to many other studies which only assessed for the first 12 months.

## Conclusions

We conclude that GnRH agonists are not an effective modality for gonadoprotection in the setting of gonadotoxic conditioning regimens for an HSCT. Further research should be done with RCTs and prospective studies in similar patient populations to corroborate our data.

## Appendices

**Table 2:** STROBE guideline Checklist of items that should be included in reports of cohort studies STROBE: STrengthening the Reporting of OBservational studies in Epidemiology

	Item No.	Recommendation
Title and abstract	1	(a) Indicate the study’s design with a commonly used term in the title or the abstract
		(b) Provide in the abstract an informative and balanced summary of what was done and what was found
Introduction		
Background/rationale	2	Explain the scientific background and rationale for the investigation being reported
Objectives	3	State-specific objectives, including any prespecified hypotheses
Methods		
Study design	4	Present key elements of study design early in the paper
Setting	5	Describe the setting, locations, and relevant dates, including periods of recruitment, exposure, follow-up, and data collection
Participants	6	(a) Give the eligibility criteria, and the sources and methods of selection of participants. Describe methods of follow-up
		(b) For matched studies, give matching criteria and number of exposed and unexposed
Variables	7	Clearly define all outcomes, exposures, predictors, potential confounders, and effect modifiers. Give diagnostic criteria, if applicable
Data sources/measurement	8	For each variable of interest, give sources of data and details of methods of assessment (measurement). Describe comparability of assessment methods if there is more than one group
Bias	9	Describe any efforts to address potential sources of bias
Study size	10	Explain how the study size was arrived at
Quantitative variables	11	Explain how quantitative variables were handled in the analyses. If applicable, describe which groupings were chosen and why
Statistical methods	12	(a) Describe all statistical methods, including those used to control for confounding
		(b) Describe any methods used to examine subgroups and interactions
		(c) Explain how missing data were addressed
		(d) If applicable, explain how loss to follow-up was addressed
		(e) Describe any sensitivity analyses
Results		
Participants	13	(a) Report numbers of individuals at each stage of study - e.g. numbers potentially eligible, examined for eligibility, confirmed eligible, included in the study, completing follow-up, and analysed
		(b) Give reasons for non-participation at each stage
		(c) Consider use of a flow diagram
Descriptive data	14	(a) Give characteristics of study participants (e.g. demographic, clinical, social) and information on exposures and potential confounders
		(b) Indicate number of participants with missing data for each variable of interest
		(c) Summarise follow-up time (e.g. average and total amount)
Outcome data	15	Report numbers of outcome events or summary measures over time
Main results	16	(a) Give unadjusted estimates and, if applicable, confounder-adjusted estimates and their precision (e.g. 95% confidence interval). Make clear which confounders were adjusted for and why they were included
		(b) Report category boundaries when continuous variables were categorized
		(c) If relevant, consider translating estimates of relative risk into absolute risk for a meaningful time period
Other analyses	17	Report other analyses done - e.g. analyses of subgroups and interactions, and sensitivity analyses
Discussion		
Key results	18	Summarise key results with reference to study objectives
Limitations	19	Discuss limitations of the study, taking into account sources of potential bias or imprecision. Discuss both direction and magnitude of any potential bias
Interpretation	20	Give a cautious overall interpretation of results considering objectives, limitations, multiplicity of analyses, results from similar studies, and other relevant evidence
Generalisability	21	Discuss the generalisability (external validity) of the study results
Other information		
Funding	22	Give the source of funding and the role of the funders for the present study and, if applicable, for the original study on which the present article is based
